# An integrated approach for SNP calling based on population of genomes

**DOI:** 10.1186/1471-2105-15-S10-P30

**Published:** 2014-09-29

**Authors:** Nam S Vo, Quang Tran, Vinhthuy Phan

**Affiliations:** 1 Department of Computer Science, University of Memphis, Memphis, TN 38152, USA; 2 Bioinformatics Program, University of Memphis, Memphis, TN 38152, USA

## Background

The identification of genetic variants such as single nucleotide polymorphisms (SNPs) is a critical step in many applications based on NGS technologies [1]. Although many SNP calling programs have been developed, it is still challenging to accurately call SNPs, especially when coverage level is low [2]. Moreover, the determination of SNPs, which is performed through many separate steps, requires a careful selection of a diverse set of tools [3,4]. This can lead to several disadvantages, for example, one cannot incorporate information from the read alignment step into the SNP calling step or vice versa to help improve accuracy of called SNPs.

## Materials and methods

We propose a novel integrated approach to detect more true SNPs while calling fewer false positives. Different from current methods that perform read alignment and SNP calling steps separately, our method combines them methodologically to improve the accuracy of SNP identification. To effectively exploit information from a population of genomes, databases of confirmed SNPs, such as dbSNP, are employed in both aligning reads to references as well as calling SNPs. This strategy allows us to develop a novel algorithm to align reads to references that can differentiate sequencing errors from SNPs.

## Results

Based on this result, the method can call SNPs accurately and effectively even with low-coverage sequencing data. Our results on simulated data show that the method is able to call SNPs with very high precision and recall rate with low-coverage datasets.

## Conclusions

With the existence of databases of confirmed SNPs for large amounts of sequenced species, our approach provides a promising method to call accurate SNP information even with low-coverage sequencing data. This approach can also help researchers facilitate the determination of SNPs by using an integrated SNP calling tool.

## References

[B1] Nielsen R Paul JS Albrechtsen A Song YS Genotype and snp calling from next-generation sequencing data Nat Rev Genet 2011 12 6 443 451 10.1038/nrg2986 21587300 PMC3593722

[B2] Yu X Sun S Comparing a few snp calling algorithms using low-coverage sequencing data BMC Bioinformatics 2013 14 274 10.1186/1471-2105-14-274 24044377 PMC3848615

[B3] Altmann A Weber P Bader D Preuss M Binder EB Müller-Myhsok B A beginners guide to snp calling from high-throughput dna-sequencing data Hum Genet 2012 131 10 1541 1554 10.1007/s00439-012-1213-z 22886560

[B4] Pabinger S Dander A Fischer M Snajder R Sperk M Efremova M Krabichler B Speicher MR Zschocke J Trajanoski Z A survey of tools for variant analysis of next-generation genome sequencing data Brief Bioinform 2014 15 2 256 278 10.1093/bib/bbs086 23341494 PMC3956068

